# Nephrotoxic drug burden among 1001 critically ill patients: impact on acute kidney injury

**DOI:** 10.1186/s13613-019-0580-1

**Published:** 2019-09-23

**Authors:** Stephan Ehrmann, Julie Helms, Aurélie Joret, Laurent Martin-Lefevre, Jean-Pierre Quenot, Jean-Etienne Herbrecht, Dalila Benzekri-Lefevre, René Robert, Arnaud Desachy, Fréderic Bellec, Gaëtan Plantefeve, Anne Bretagnol, Auguste Dargent, Jean-Claude Lacherade, Ferhat Meziani, Bruno Giraudeau, Elsa Tavernier, Pierre-François Dequin, Clinical research in intensive care and sepsis-Trial group for global evaluation and research in sepsis (CRICS-TRIGGERSEP network)

**Affiliations:** 1 INSERM CIC 1415, CHRU de Tours, Médecine intensive réanimation, 2, Bd Tonnellé, 37044 Tours Cedex 9, France; 2 Université de Tours, faculté de médecine, Tours, France; 3 ImmunoRhumatologie Moléculaire, INSERM UMR_S1109, LabEx TRANSPLANTEX, FHU OMICARE, FMTS, Université de Strasbourg, Strasbourg, France; 4 Réanimation polyvalente, CHD de Vendée, La Roche-sur-Yon, France; 5 Department of Intensive Care, François Mitterrand University Hospital, Dijon, France; 6 Lipness Team, INSERM Research Center LNC-UMR1231 and LabExLipSTIC, University of Burgundy, Dijon, France; 7 INSERM CIC 1432, Clinical Epidemiology, University of Burgundy, Dijon, France; 8 Réanimation médicale, Hôpitaux universitaires de Strasbourg, Hôpital Hautepierre, Strasbourg, France; 9 Médecine intensive réanimation, CHR d’Orléans, Orléans, France; 10 Réanimation médicale, CHU de Poitiers, Poitiers, France; 11 Réanimation polyvalente, CH d’Angoulême, Angoulême, France; 12 Réanimation, CH de Montauban, Montauban, France; 13 Réanimation polyvalente, CH Victor Dupouy, Argenteuil, France; 14 Inserm CIC 1415, CHRU de Tours, Tours, France; 15 Médecine Intensive Réanimation, Nouvel Hôpital Civil, Hôpitaux universitaires de Strasbourg, Strasbourg, France; 16 INSERM UMR 1260, Regenerative Nanomedicine (RNM), FMTS, Université de Strasbourg, Strasbourg, France

**Keywords:** Renal insufficiency [MeSH], Kidney tubular necrosis, Acute [MeSH], Contrast media [MeSH], Aminoglycosides [MeSH], Vancomycin [MeSH], Diuretics [MeSH], Intensive-care units [MeSH]

## Abstract

**Background:**

Nephrotoxic drug prescription may contribute to acute kidney injury (AKI) occurrence and worsening among critically ill patients and thus to associated morbidity and mortality. The objectives of this study were to describe nephrotoxic drug prescription in a large intensive-care unit cohort and, through a case–control study nested in the prospective cohort, to evaluate the link of nephrotoxic prescription burden with AKI.

**Results:**

Six hundred and seventeen patients (62%) received at least one nephrotoxic drug, among which 303 (30%) received two or more. AKI was observed in 609 patients (61%). A total of 351 patients were considered as cases developing or worsening AKI a given index day during the first week in the intensive-care unit. Three hundred and twenty-seven pairs of cases and controls (patients not developing or worsening AKI during the first week in the intensive-care unit, alive the case index day) matched on age, chronic kidney disease, and simplified acute physiology score 2 were analyzed. The nephrotoxic burden prior to the index day was measured in drug.days: each drug and each day of therapy increasing the burden by 1 drug.day. This represents a semi-quantitative evaluation of drug exposure, potentially easy to implement by clinicians. Nephrotoxic burden was significantly higher among cases than controls: odds ratio 1.20 and 95% confidence interval 1.04–1.38. Sensitivity analysis showed that this association between nephrotoxic drug prescription in the intensive-care unit and AKI was predominant among the patients with lower severity of disease (simplified acute physiology score 2 below 48).

**Conclusions:**

The frequently observed prescription of nephrotoxic drugs to critically ill patients may be evaluated semi-quantitatively through computing drug.day nephrotoxic burden, an index significantly associated with subsequent AKI occurrence, and worsening among patients with lower severity of disease.

## Background

Acute kidney injury (AKI) frequently occurs in critically ill patients and is associated with high morbidity and mortality in the short and long term [1–4]. AKI is of multifactorial origin in most critically ill patients [5, 6]. Beyond fluid loading and general perfusion pressure maintenance, research failed until now to identify effective specific interventions to prevent AKI [7–9]. Pathophysiology of AKI is complex, involving multiple pathways including inflammation, hypoxia, and oxidative stress, leading to renal cellular injury [4–6]. Drug toxicity acting through several of those pathways is a potentially modifiable risk factor for AKI [10–14]. Indeed, given the high risk of short-term death, physicians may consider the overall benefit/risk ratio in favor of giving drugs despite their potential for damaging the kidneys. Nevertheless, even modest kidney dysfunction is associated with adverse patients’ outcome [15, 16]. The multifactorial nature of AKI and the numerous nephrotoxic drugs potentially delivered to critically ill patients make it very challenging to delineate attributable risk of AKI to specific drugs. Epidemiologic studies identified drug toxicity as a contributing factor in 15–25% of AKI cases [17–20]. Conversely, studies investigating specific contribution of individual drugs to AKI either did not identify any attributable risk, showed conflicting results, or confirmed clinical relevance of toxicity [21–26]. Large AKI epidemiology studies did not specifically evaluated nephrotoxic causes of AKI which represents a important limit. Conversely, studies specifically addressing nephrotoxicity were focused on one drug class only and did not evaluate the global nephrotoxic burden patients experience. To the best of our knowledge, the literature is lacking a large multicenter descriptive study of nephrotoxic prescription pattern in ICU patients evaluating the link between nephrotoxicity and AKI. Such knowledge is not only descriptive or academic in the sense that identifying nephrotoxic prescription burden and its impact on AKI may lay the foundations for interventional trials minimizing this potential aggression. Tackling drug toxicity represents a unique and simple mean to actively impact AKI in the ICU. The objective of this study was to give a large multicenter description of nephrotoxic drug prescription in ICUs, to semi-quantitatively evaluate the nephrotoxic burden experienced by critically ill patients, and to investigate its relationship with AKI.

## Methods

This multicenter prospective descriptive cohort study was conducted in ten ICUs in France. Reporting follows the STROBE guidelines for observational studies (http://www.equator-network.org). The study lasted 8 weeks, divided into 2 inclusion periods of 4 weeks in each participating center: one in the winter and one in the subsequent summer in the years 2014–2015. All adult patients were included except patients who underwent renal replacement therapy within the past week, on a chronic basis or the day of ICU admission and patients with expected very short stay (< 48 h).

Data were prospectively collected in an electronic clinical record file. None of the participating centers had a clinical pharmacist involved at the bedside. Aside of demographic data, admission diagnosis, co-morbidities, and severity of illness (simplified acute physiology score 2 (SAPS2) [27]) exposition to nephrotoxic medication and occurrence of AKI were recorded.

Nephrotoxic drug delivery was recorded daily during the first 7 days in the ICU, using a predefined list of drugs based on a literature review performed prior to study initiation (Table 1) [10–12, 28]. Beta-lactam antibiotics were recorded as potentially nephrotoxic only when given at high dose for neurologic infections or endocarditis.

**Table 1:** Potential nephrotoxic drugs recorded

Cardiovascular drugs
Diuretics: thiazide diuretics, furosemide, bumetanide, spironolactone
Angiotensin-converting enzyme inhibitors
Angiotensin II receptor antagonist
Antibiotics
Vancomycin
Aminoglycosides: amikacin, gentamicin, netilmicin
High-dose beta-lactams, i.e., central nervous system or endocarditis dosage (e.g., amoxicillin 100 mg/kg or higher)
Rifampicin
Sulfadiazine
Cotrimoxazole
Antiviral agents
Nucleosidic inhibitors: acyclovir, adefovir, cidofovir, tenofovir, indinavir
Foscarnet
Antifungal agents
Amphotericin B
Voriconazole
Immunosuppressors/chemotherapy
Cisplatin
Methotrexate
Ciclosporin
Tacrolimus, everolimus
Mycophenolate mofetil
Immunoglobulins
Other
Non-steroidal anti-inflammatory drugs (including acetylsalicylic acid)
Hydroxy-ethyl starch
Mannitol
Lithium
Zoledronic acid
Iodinated contrast media
Gadolinium

AKI incidence was recorded over the same period, based on urine output and serum creatinine concentration according to the Kidney Disease Improving Global Outcome classification (KDIGO), see Additional file 1 for details [29].

Patients’ outcome was further evaluated recording renal replacement therapy requirement, ICU, and hospital lengths of stay and mortalities.

### Statistical analysis

Quantitative variables were expressed as means and standard deviations or medians and quartiles, and qualitative variables as counts and percentages. Descriptive statistics were used to characterize the prescription of nephrotoxic drugs. No missing data imputation was performed. Global nephrotoxic exposure of patients was quantified as nephrotoxic drug.days, enabling a semi-quantitative assessment of the overall nephrotoxic burden experienced by a patient, as defined a priori. With this method, each day a patient receives one nephrotoxic drug is allocated 1 point of nephrotoxic burden. Patients receiving more than one nephrotoxic drug are allocated the number of points equal to the number of nephrotoxic drugs received for that given day. For example, a patient receiving a 3 day course of acyclovir is allocated a nephrotoxic burden drug.days value of 3; a patient receiving a 3 days course of vancomycin associated with an aminoglycoside will be allocated a nephrotoxic burden drug.days value of 6. This semi-quantitative evaluation of nephrotoxic burden may overlook some differences between drugs with higher or lower kidney toxicity, but represents a pragmatic choice, easy to implement at the bedside.

To study the association between the ICU nephrotoxic burden and AKI, a case–control analysis was nested within the prospective cohort. Cases were patients who experienced AKI worsening between the second and the seventh day of the ICU stay as compared to their admission to the unit (increase in KDIGO classification stage). Thus, cases were patients admitted to the ICU without AKI (KDIGO stage 0), who subsequently developed KDIGO stages 1, 2, or 3 AKI and patients admitted with already ongoing AKI (KDIGO stages 1 or 2), who subsequently developed KDIGO stage 2 and/or 3 AKI. Patients admitted to the ICU with already ongoing KDIGO stage 3 AKI were not considered as cases, because the presence of maximal AKI right at admission prevented analysis of worsening. Controls were patients without AKI worsening during the first 7 days of the ICU stay (i.e., no AKI or same KDIGO stage as at admission). Cases were matched 1:1, on age (5 years margin), chronic kidney disease, and admission SAPS2 (5 points margin) [27], with control patients alive the index day the case patient developed AKI worsening (see Additional file 1: Figure S1). The exposure history of each matched case and control was considered up to the index day at which the corresponding case became a case. The nephrotoxic burden in drug.days was compared between cases and controls using conditional logistic regression accounting for matching. This cumulative sampling was performed with replacement of the controls, and, to take into account the non-independent nature of the data, a weighting on inverse of frequency was implemented [30, 31]. Results were expressed as an odds ratio (OR) of nephrotoxic burden with its 95% confidence interval (CI_95_).

Sensitivity analyses were performed: (1) with cases and controls, defined as patients without AKI at admission who developed or did not develop it during the first 7 days in the ICU. (2) Among patients with admission SAPS2 below and above the cohort median value. (3) Matching cases and controls without replacement and using wider matching margins (age ± 10 years; admission SAPS2 ± 10 points) to assess the consistency of the matching procedure. Furthermore, analysis was performed adjusting the conditional logistic regression on the presence or absence of sepsis the first day of ICU admission.

Impact of AKI on patient survival was evaluated using a Cox model using time-dependent covariates, adjusted on admission SAPS2.

The planned sample size was 1000 patients, expecting 300 cases and 300 controls. This sample size enabled to observe a significant odds ratio of 1.70 with a power of 80% and a type I error of 5% with 20% of the patients receiving at least one nephrotoxic drug. Analyses were performed using R [R Development Core Team (2008). R: A language and environment for statistical computing. R Foundation for Statistical Computing, Vienna, Austria] and SAS software (SAS Institute Inc., Cary, NC, USA).

## Results

Characteristics of the 1001 patients included are presented in Table 2. No missing data were observed on the main variables of interest. Six hundred and seventeen patients (62%) received at least one nephrotoxic drug during their first 7 days in the ICU. Among those, 184 (30%) received two and 119 (19%) received three or more different nephrotoxic drugs. Most frequent nephrotoxic drugs received in the ICU are presented in Table 3.

**Table 2:** Main patients’ characteristics

Variables	*N* = 1001 patients
Female gender	362 (36%)
Age (years)	65 ± 16
Main admission diagnosis	
De novo acute respiratory failure	182 (18%)
Coma, seizure	153 (15%)
Sepsis and septic shock	150 (15%)
Chronic respiratory failure exacerbation	139 (14%)
Cardiac arrest	95 (10%)
Hemorrhagic and hypovolemic shock	34 (3%)
Cardiogenic shock	31 (3%)
Post-operative monitoring	27 (3%)
Acute renal failure	14 (1%)
Other	175 (18%)
Admission origin	
Emergency department	388 (39%)
Ward	433 (43%)
Home	179 (18%)
Simplified acute physiology score 2	47 ± 20
Invasive mechanical ventilation	617 (62%)
Co-morbidities	
Arterial hypertension	513 (51%)
Diabetes mellitus	236 (24%)
Chronic respiratory failure	206 (21%)
Ischemic heart disease	161 (16%)
Chronic heart failure	122 (12%)
Peripheral artery disease	115 (12%)
Chronic kidney disease	96 (10%)
Cirrhosis	47 (5%)
Outcomes	
Intensive-care unit mortality	235 (24%)
Hospital mortality	285 (29%)
Intensive-care unit lengths of stay (days)	4 [2, 7]
Hospital lengths of stay (days)	10 [4, 21]

Qualitative variables are presented as count (percentage) and quantitative variables as mean ± standard deviation or median [interquartile range]

**Table 3:** Nephrotoxic drugs most frequently prescribed among exposed patients

Drugs	*n* = 617
Iodinated contrast media	154 (25%)
Diuretics	356 (58%)
Loop diuretics	346 (97%)
Thiazide diuretics	18 (5%)
Potassium sparing diuretics	10 (3%)
Antibiotics	227 (37%)
Vancomycin	77 (34%)
Aminoglycosides	139 (61%)
High-dose beta-lactams	57 (25%)
Sulfamethoxazole trimethoprim	25 (11%)
Rifampicin	12 (5%)
Antiviral agents	58 (9%)
Acyclovir	36 (62%)
Other	22 (38%)
Antifungal agents	27 (4%)
Amphotericin B	9 (33%)
Voriconazole	12 (44%)
Other	6 (22%)
Renin–angiotensin–aldosterone antagonists	96 (16%)
Hydroxy-ethyl-starch	2 (< 0.5%)
Mannitol	5 (< 0.5%)
Non-steroidal anti-inflammatory drugs (including acetylsalicylic acid)	84 (14%)
Immunosuppressants and chemotherapy	16 (3%)
Other	56 (9%)

Data are presented as counts (percentage). Percentages are calculated among patients receiving at least one nephrotoxic prescription during the first 7 days in the intensive-care unit and within each drug class

**Fig. 1:**
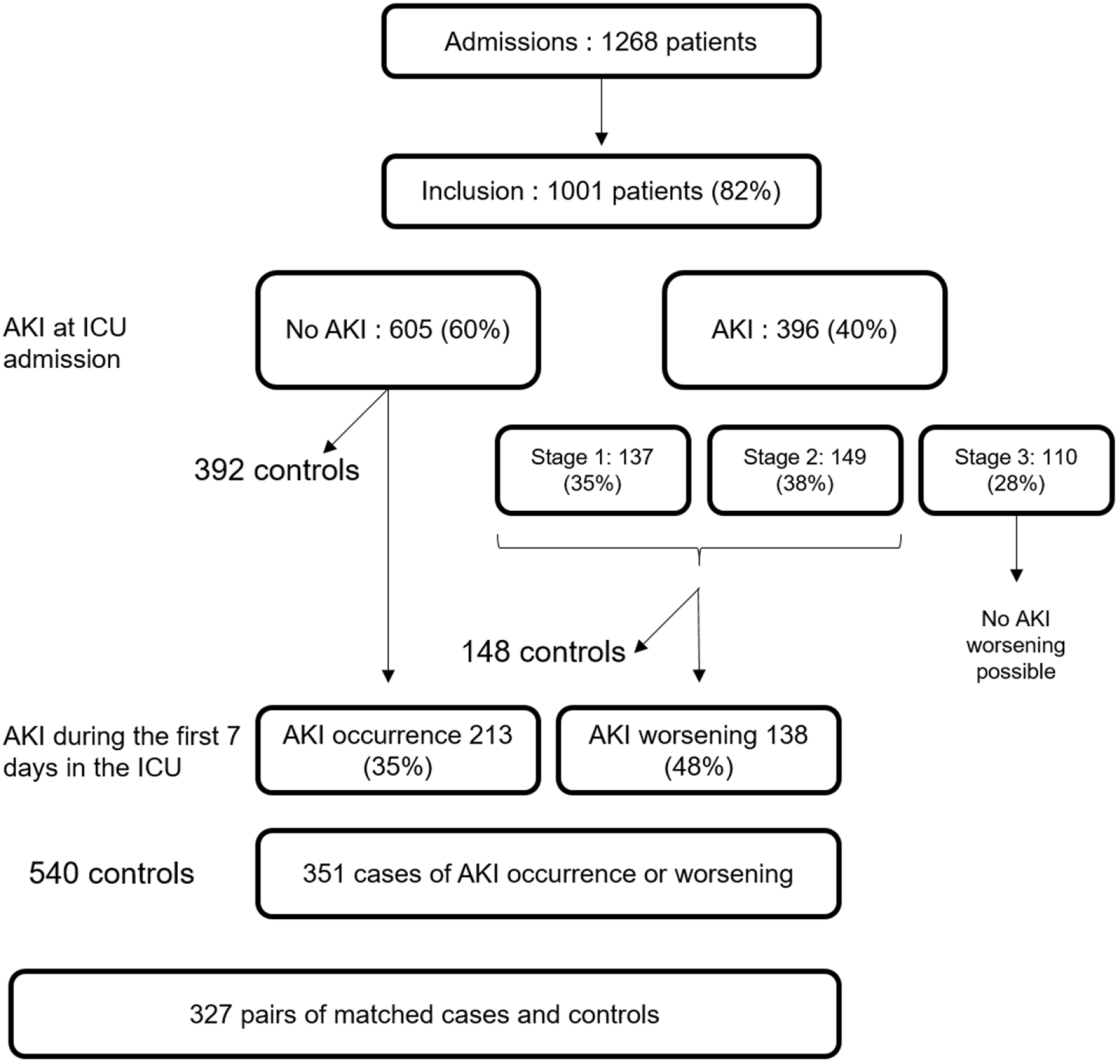
Study flowchart

AKI occurrence is depicted in the study flow chart (Fig. 1): 396 (40%) patients experienced AKI the day of ICU admission. Among the 286 patients with KDIGO stage 1 or 2 at ICU admission, 138 (48%) experienced AKI worsening with development of stage 2 and/or 3 of the KDIGO classification within 7 days. Thus, a total of 351 (35%) patients experienced either occurrence or worsening of AKI in the ICU (cases), subsequent to potential nephrotoxic drug prescription in the unit. The median day of occurrence or worsening was day 2 (interquartile range days 2–4). Forty-nine patients (5%) underwent renal replacement therapy. AKI occurrence as well as its worsening during the first 7 days in the ICU were independent risk factors of death in the hospital (death hazard ratio (HR) of, respectively, 1.45, IC_95_ [1.06–1.98], *p* = 0.021 and 2.00, IC_95_ [1.38–2.88], *p* < 0.001) for AKI after adjusting for admission SAPS2.

Of note before ICU admission, a large proportion of patients received nephrotoxic medication: 644 (64%) received at least one nephrotoxic medication within the 48 h of ICU admission, either as chronic maintenance therapy (n = 530, 53%), or as a newly initiated prescription (n = 297, 30%).

**Fig. 2:**
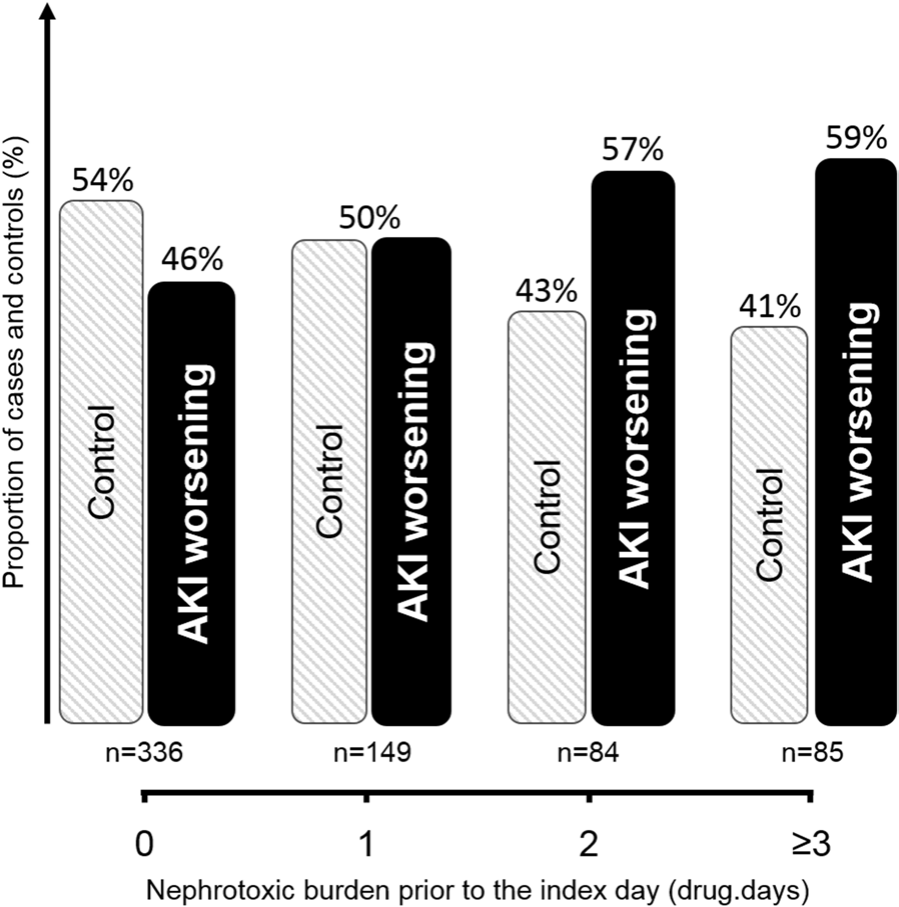
Proportion of cases and control patients among the 327 matched pairs according to increasing nephrotoxic burden prior to the index day (see text for definition). The proportion of cases of acute kidney injury (AKI) occurrence/worsening increased and the proportion of controls decreased with increasing nephrotoxic burden, thus supporting a dose–response relationship

**Fig. 3:**
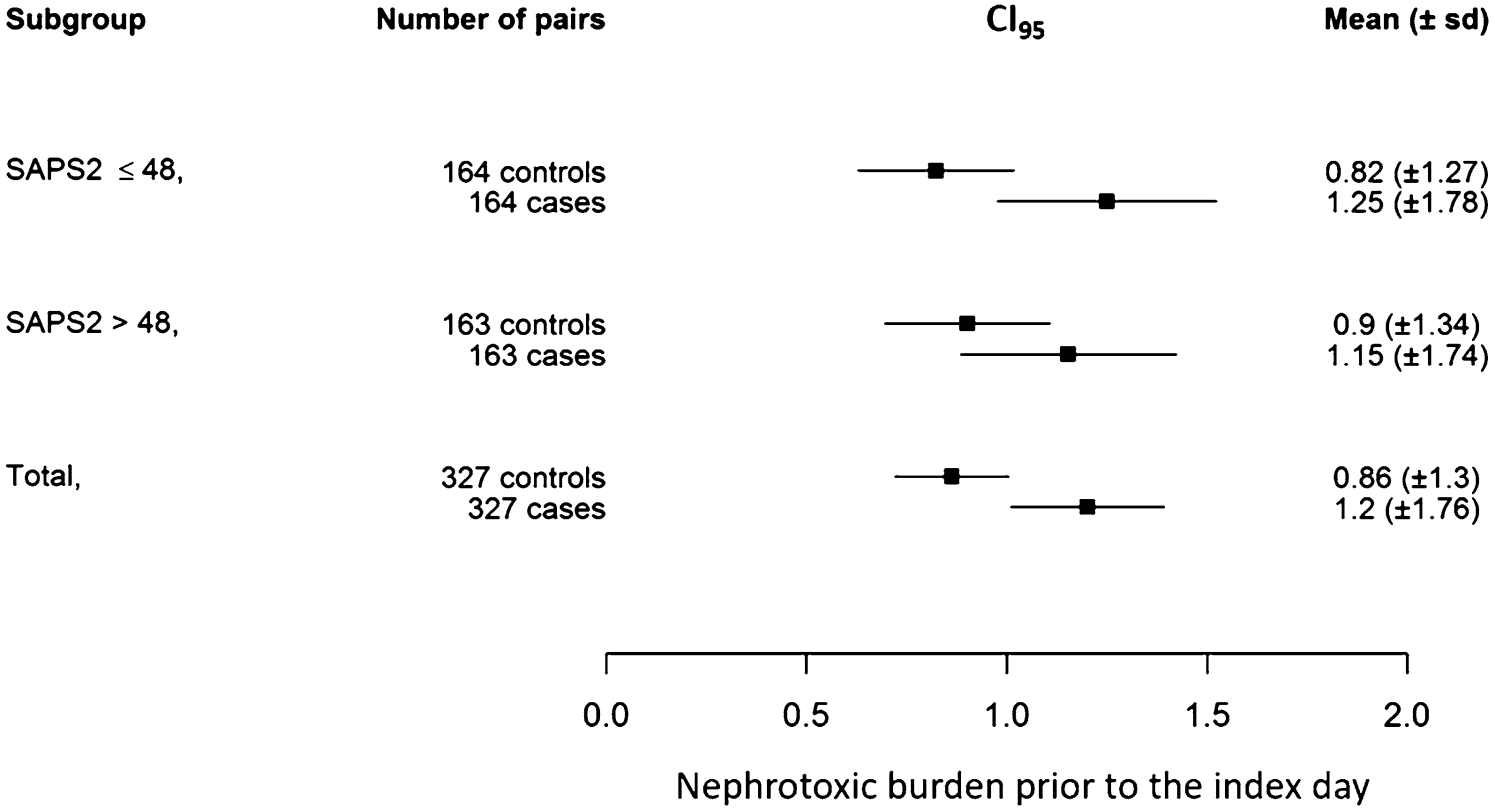
Severity of disease sensitivity analysis. Squares represent mean values of the nephrotoxic burden prior to the index day with error bars indicating 95% confidence interval (IC_95_). Whereas nephrotoxic burden prior to the index day was higher among cases than controls in the overall population [bottom line: associated odds ratio (OR) 1.20, IC_95_ 1.04–1.38] and among patients with a simplified acute physiology score 2 (SAPS2) below the median value of the population (SAPS2 = 48) indicating lower severity of disease (upper line, associated OR 1.26 IC_95_ 1.03–1.56), no such association was observed among the most severe patients (SAPS2 > 48, middle line, associated OR 1.13 IC_95_ 0.92–1.37)

Among the 351 cases, 327 could be matched to 327 controls (representing 195 distinct patients due to matching with replacement). Main characteristics of cases, controls, and patients not included in the matched cohort are presented in the Additional file 1: Table S1. The proportion of cases of AKI worsening as compared to control patients increased with increasing nephrotoxic burden prior to the index day, exhibiting a dose–response relationship (Fig. 2). Conditional logistic regression among matched cases and controls showed a significant increased risk of AKI development/worsening with increasing nephrotoxic burden prior to the index day: OR 1.20 IC_95_ [1.04–1.38] (*p* = 0.015) in the whole matched population. Nephrotoxic burden prior to the index day was 0.86 ± 1.30 drug.days among control patients and 1.20 ± 1.76 drug.days among cases (median and interquartile ranges of, respectively, 0 [0; 1] and 1 [0; 2] (Fig. 3). Among cases and controls, AKI worsening was independently associated with hospital mortality (HR = 3.17 [1.81; 5.54] *p* < 0.001, adjusted on admission SAPS2). Among the most frequently prescribed nephrotoxic drugs, namely iodinated contrast media, diuretics, and antibiotics, the relative contribution of each drug to the difference in nephrotoxic burden observed between cases and controls is presented in the supplemental digital content (Additional file 1: Table S2).

**Fig. 4:**
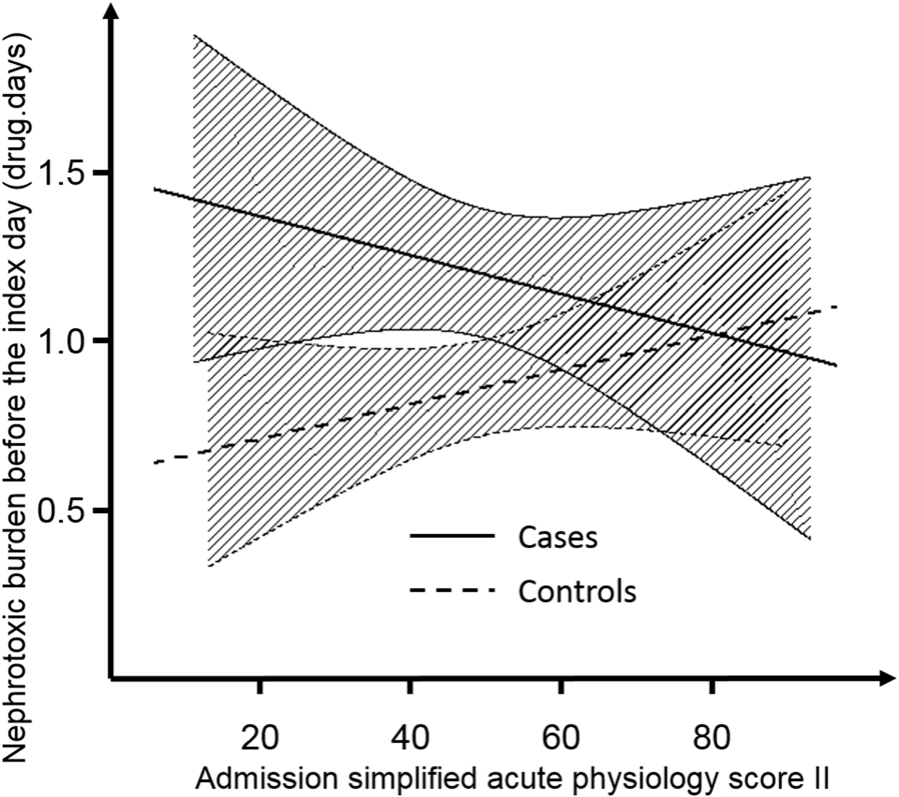
Nephrotoxic burden according to severity of disease. Whereas an increase in nephrotoxic burden before the index day (see text for definition) was observed among control patients not developing or worsening acute kidney injury in the intensive-care unit (control patients) within increasing severity, a decrease was observed for cases of acute kidney injury occurrence or worsening

Sensitivity analysis according to severity of disease showed that the link between semi-quantitative evaluation of nephrotoxic burden and AKI was apparent in the patients with lower severity of disease (admission SAPS2 below the median value of the population of 48): OR of 1.26 IC_95_ [1.03–1.56], *p* = 0.026. Among the sickest patients (admission SAPS2 higher than 48), the association did not reach statistical significance: OR 1.13 IC_95_ [0.92–1.37], *p* = 0.245 (Fig. 3). Nephrotoxic burden among cases of AKI occurrence/worsening and of controls was plotted according to severity of disease in Fig. 4. Interestingly, whereas nephrotoxic burden steadily increased with severity of disease among control patients, the opposite was observed for cases of acute kidney injury occurrence/worsening (Fig. 4). Sensitivity analysis performing matching without replacement of controls showed results similar to the primary analysis: OR 1.19 IC_95_ [1.12–1.27] (*p* = 0.004), as well as using wider matching margins (data not shown). Sensitivity analysis among the subgroup of patients admitted without AKI showed results similar to the whole population in favor of a significant impact of nephrotoxic burden on AKI occurrence: 192 cases matched with 192 controls, OR of nephrotoxic burden for AKI occurrence 1.37 IC_95_ [1.12–1.69] (*p* = 0.003). Adjusting the conditional logistic regression on sepsis diagnosis, the day of ICU admission showed similar results to the main analysis: OR = 1.18 IC_95_ [1.02; 1.37], *p* = 0.023. Specifically evaluating diuretics, they were more frequently prescribed among cases of AKI occurrence or worsening (23%) than among control patients (16%), *p* < 0.001. No interaction (*p* = 0.77) was observed with the timing of diuretic administration in the course of the ICU stay (first 3 days versus only later on).

## Discussion

Both prescription of nephrotoxic drugs and AKI appeared very common among critically ill patients. Adjusting for confounders, the former, as semi-quantitatively evaluated through the drug.days nephrotoxic burden, was independently associated with the latter, overall, and among the patients with lower severity of disease. As AKI was confirmed to be an independent mortality risk factor, one may speculate on a negative link between nephrotoxic drug administration and patient outcome, a hypothesis to be tested in an interventional trial. Austin Bradford Hill proposed consensual criteria to assess a potential causal relationship between exposure and disease [32]. Whereas *plausibility*, *analogy,* and *coherence* may be assumed based on preclinical data, the present work adds evidence in favor of a causal link among several other Hill criteria. (1) *Temporality* was taken into account as nephrotoxic burden was considered only before the index day among cases and controls. (2) *Consistency* is reinforced by the large multicentric nature and the sensitivity analyses of the present work. However, given that nephrotoxic burden, as quantified in the present study, is a relatively novel approach, it should be further validated among independent patient cohorts. (3) *Biological gradient* (i.e., dose–response relationship) was observed with an increasing risk of AKI for increasing nephrotoxic burden (Fig. 2). (4) *Specificity* of the link between nephrotoxic burden and AKI may be assumed as case–control matching accounts for known and potentially for unknown confounders. (5) *Strengths of the association* may be considered important with an odd ratio of 1.20 for each 1 drug.day increase in nephrotoxic burden, i.e., one additional nephrotoxic drug delivered or one additional day of therapy with such a drug (e.g., 3.60 OR for a 3-day course of aminoglycosides). Last but not least, Hill criteria, i.e., *clinical experimental evidence*, cannot be assumed given the observational design of the present work. Nevertheless, the present observations give important information to design an interventional trial on the topic. A trial evaluating a reduced nephrotoxic burden bundle (restricting prescription indications, dose, and duration) to prevent AKI and associated poor outcome would need to specifically address iodinated contrast media, loop diuretics, and aminoglycosides, the main contributors to excess nephrotoxic burden among AKI cases (Additional file 1: Table S2, Additional digital content), and target patients with lower severity of disease, early in the ICU stay. Furthermore, evaluation of the impact of nephrotoxic burden on AKI recovery may deserve evaluation. In the meanwhile, monitoring nephrotoxic burden by clinical pharmacists and/or the use of electronic alerts may be interesting means to make clinicians aware of the nephrotoxic burden imposed to patients and its potentially deleterious consequences. The simplicity of drug.days nephrotoxic burden calculation may enable to envision relatively easy clinical implementation.

Our results are consistent with interventional studies showing an increased incidence of AKI when aminoglycosides are associated with beta-lactam antibiotics to treat sepsis compared to monotherapy [26]. Among pediatric critically ill patients, nephrotoxic drug prescription was associated with AKI, with the same drugs involved and observation of a similar dose–response relationship as observed in the present work [33].

Conversely, our results may seem in contradiction with evidence showing a lack of association between some potential nephrotoxic drugs and AKI, e.g., iodinated contrast media [23]. The present study assumes an additive nephrotoxic effect, thus revealing a total clinically relevant toxicity which may not be apparent when evaluating drugs separately. This additive toxicity hypothesis may be challenged given the heterogeneity of toxicity mechanisms. However, concerning iodinated contrast media toxicity for example, most experimental evidence was observed in two hit models, e.g., hypovolemia (such as may be induced by diuretics) prior to contrast administration, supporting additive phenomenon [34]. In a large case–control study, observing an association between nephrotoxic drug delivery and AKI similar to the present work, more than half of the patients experiencing AKI received several nephrotoxic medications supporting additive toxic phenomenon [35].

The present study has important limitations. First, despite matching, one cannot exclude the presence of some residual confounders. Only an interventional trial may definitively prove a causal relationship between nephrotoxic drug prescription and AKI occurrence. Second, one may discuss the list of nephrotoxic medications recorded. In particular for immune-allergic mechanisms, the potential drug list may be endless. Likewise, growing evidence in favor of nephrotoxicity of the combination of piperacillin–tazobactam with vancomycin emerged since study design [36]. However, only 77 patients (8% of the cohort) received vancomycin, and thus, the combination of piperacillin–tazobactam with vancomycin at best concerned a minority of patient unlikely to alter overall results. Recording potential nephrotoxicity of beta-lactam antibiotics only when given at high dose also represents a limitation. Indeed, aside of dose-related high urinary drug concentration representing a well-established risk factor, low pH and low urine output may also favor crystalluria, sometimes observed in patients receiving normal antibiotic doses [37–39]. Conversely, the nephrotoxic potential of some drugs recorded may be debated such as acetylsalicylic acid or gadolinium recorded in a minority of patients [40]. However, one cannot exclude that drugs with a low potential nephrotoxicity may participate to the additive nephrotoxic burden. Given the lack of consensus concerning the drugs with the highest nephrotoxic potential in the ICU setting, the choice was made of a relatively large drug panel to be recorded. Third, various mechanisms of nephrotoxicity may come into play for a given drug, e.g., diuretics may induce AKI through hypovolemia and/or interstitial nephritis. Indeed, in the setting of fluid overload and congestive heart failure, diuretics may even prove beneficial on overall cardiovascular dynamics and thus on renal function. However, precise effective volemia is difficult to measure and could not be recorded in the present large-scale study; trials evaluating diuretics as a mean to prevent AKI occurrence or worsening in the critical care setting were negative [9]. Therefore, like others [33], we made the pragmatic choice of considering the potential deleterious effect of diuretics, mediated through induced hypovolemia, as predominant in the population under study. Finally, when calculating nephrotoxic burden, the same weight was attributed to all drugs. Weighting the contribution of each drug according to its nephrotoxic potential may theoretically be an appealing method to overcome this limit; however, set weighting coefficients would be highly debatable given the literature scarcity. In the same line, the administered dose and potential overdosing were not taken into account. This pragmatic choice represents a limitation of the study, but the simplicity of the nephrotoxic burden concept evaluated here may facilitate its adoption by clinicians. Indeed, physicians may easily calculate or be alerted of a nephrotoxic drug burden increase above a set threshold. Withholding one nephrotoxic drug prescription or 1 day of therapy with such a drug may represent a meaningful action for physicians to keep nephrotoxic burden low. Such clinical strategies will need to be investigated, defining thresholds and prescription scenarios.

## Conclusions

The frequently observed prescription of nephrotoxic drugs to critically ill patients may be evaluated semi-quantitatively through computing drug.day nephrotoxic burden, an index significantly associated with subsequent AKI occurrence and worsening among patients with lower severity of disease. Those results call for the design of interventional trials prospectively testing strategies to reduce the nephrotoxic drug burden in the ICU.

## Supplementary information

**Additional file 1: Figure S1.** Acute kidney injury (AKI) worsening cases and control matching. **Table S1.** Main characteristics of matched and unmatched patients. **Table S2.** Relative contribution individual drugs to the difference in nephrotoxic burden experienced by cases and controls.

## Data Availability

The data sets used and/or analyzed during the current study are available from the corresponding author on reasonable request.

## Abbreviations

AKI: acute kidney injury
CI_95_: 95% confidence interval
HR: hazard ratio
KDIGO: kidney disease improving global outcome classification
OR: odds ratio
SAPS2: simplified acute physiology score 2

## References

[1] Joannidis M, Metnitz B, Bauer P, Schusterschitz N, Moreno R, Druml W, Metnitz PG (2009). Acute kidney injury in critically ill patients classified by AKIN versus RIFLE using the SAPS 3 database. Intensive Care Med.

[2] Waara ST, Pettilä V, Kaukonen KM, Bendel S, Korhonen AM, Bellomo R, Reinikainen M (2014). The attributable mortality of acute kidney injury: a sequentially matched analysis. Crit Care Med.

[3] Dettmer MR, Damuth E, Zarbiv S, Mitchell JA, Bartock JL, Trzeciak S (2017). Prognostic factors for long-term mortality in critically ill patients treated with prolonged mechanical ventilation: a systematic review. Crit Care Med.

[4] Chawla LS, Eggers PW, Star RA, Kimmel PL (2014). Acute kidney injury and chronic kidney disease as interconnected syndromes. N Engl J Med.

[5] Molitoris BA (2014). Therapeutic translation in acute kidney injury: the epithelial/endothelial axis. J Clin Invest.

[6] Wan L, Bagshaw SM, Langenberg C, Saotome T, May C, Bellomo R (2008). Pathophysiology of septic acute kidney injury: what do we really know?. Crit Care Med.

[7] Darmon M, Ostermann M, Cerda J, Diopoulos MA, Forni L, Hoste E (2017). Diagnostic work-up and specific causes of acute kidney injury. Intensive Care Med.

[8] Badin J, Boulain T, Ehrmann S, Skarzynski M, Bretagnol A, Buret J (2011). Relation between mean arterial pressure and renal function in the early phase of shock: a prospective, explorative cohort study. Crit Care.

[9] Joannidis M, Druml W, Forni LG, Groeneveld ABJ, Honore PM, Hoste E (2017). Prevention of acute kidney injury and protection of renal function in the intensive care unit: update 2017: expert opinion of the working group on prevention, AKI section, European society of intensive care medicine. Intensive Care Med.

[10] Bentley ML, Corwin HL, Dasta J (2010). Drug-induced acute kidney injury in the critically ill adult: recognition and prevention strategies. Crit Care Med.

[11] Taber S, Pasko DA (2008). The epidemiology of drug-induced disorders: the kidney. Expert Opin Drug Saf.

[12] Schetz M, Dasta J, Goldstein S, Golper T (2005). Drug-induced acute kidney injury. Curr Opin Crit Care.

[13] Perazella MA (2012). Drug use and nephrotoxicity in the intensive care unit. Kidney Int.

[14] Goldstein SL (2016). Medication-induced acute kidney injury. Curr Opin Crit Care..

[15] Lassnigg A, Schmidlin D, Mouhieddine M, Bachmann LM, Druml W, Bauer P (2004). Minimal changes of serum creatinine predict prognosis in patients after cardiothoracic surgery: a prospective cohort study. J Am Soc Nephrol.

[16] Nin N, Lombardi R, Frutos-Vivar F, Esteban A, Lorente JA, Ferguson ND (2010). Early and small changes in serum creatinine concentration are associated to mortality in mechanically ventilated patients. Shock.

[17] Uchino S, Kellum JA, Bellomo R, Doig GS, Morimatsu H, Morgera S (2005). Acute renal failure in critically ill patients: a multinational, multicenter study. JAMA.

[18] Mehta RL, Pascual MT, Soroko S, Savage BR, Himmelbarb J, Ikizler TA (2004). Spectrum of acute renal failure in the intensive care unit: the PICARD experience. Kidney Int.

[19] Hoste EA, Bagshaw SM, Bellomo R, Cely CM, Colman R, Cruz DN (2015). Epidemiology of acute kidney injury in critically ill patients: the multinational AKI-EPI study. Intensive Care Med.

[20] Herrera-Gutiérrez ME, Seller-Pérez G, Sánchez-Izquierdo-Riera JA, Maynar-Moliner J (2013). Prevalence of acute kidney injury in intensive care units: the “COrte de prevalencia dedisFunción RenAl y DEpuración en criticos” point-prevalence multicenter study. J Crit Care.

[21] Oliveira JFP, Silva CA, Barbieri CD, Oliveira GM, Zanetta DM, Burdmann EA (2009). Prevalence and risk factors for aminoglycoside nephrotoxicity in intensive care units. Antimicrob Agents Chemother.

[22] Lakhal K, Ehrmann S, Robert-Edan V (2017). Iodinated contrast medium renal toxicity: the phantom menace of much ado about nothing?. Crit Care Med.

[23] Ehrmann S, Quartin A, Hobbs BP, Robert-Edan V, Cely C, Bell C (2017). Contrast-associated acute kidney injury in the critically ill: systematic review and Bayesian meta-analysis. Intensive Care Med.

[24] Lacave G, Caille V, Bruneel F, Palette C, Legriel S, Grimaldi D (2017). Incidence and risk factors of acute kidney injury associated with continuous intravenous high-dose vancomycin in critically ill patients: a retrospective cohort study. Medicine (Baltimore).

[25] Picard W, Bazin F, Clouzeau B, Bui HN, Soulat M, Guilhon E (2014). Propensity-based study of aminoglycoside nephrotoxicity in patients with severe sepsis or septic shock. Antimicrob Agents Chemother.

[26] Paul M, Lador A, Grozinsky-Glasberg S, Leibovici L (2014). Beta lactam antibiotic monotherapy versus beta lactam-aminoglycoside antibiotic combination therapy for sepsis. Cochrane Database Syst Rev.

[27] Le Gall JR, Lemeshow S, Saulnier F (1993). A new simplified acute physiology score (SAPS II) based on a European/north American multicenter study. JAMA.

[28] De Broe ME, Porter GA (2008). Clinical nephrotoxins-renal injury from drugs and chemicals.

[29] Kellum JA, Lameire N, Aspelin P, Barsoum RS, Burdmann EA, Goldstein SL, Herzog CA, Joannidis M, Kribben A, Levey AS, MacLeod AM, Mehta RL, Murray PT, Naicker S, Opal SM, Schaefer F, Schetz M, Uchino S (2012). Kidney disease: Improving global outcomes (KDIGO) acute kidney injury work group. KDIGO clinical practice guideline for acute kidney injury. Kidney Int Suppl.

[30] Kelsey JL, Whittemore AS, Evans AS, Douglas Thompson W (1996). Methods in observational epidemiology.

[31] Stuart EA (2010). Matching methods for causal inference: a review and a look forward. Stat Sci.

[32] Hill AB (1965). The environment and disease: association or causation?. Proc R Soc Med.

[33] Slater MB, Gruneir A, Rochon PA, Howard AW, Koren G, Parshuram CS (2017). Identifying high-risk medications associated with acute kidney injury in critically ill patients: a pharmacoepidemiologic evaluation. Paediatr Drugs.

[34] Persson PB, Hansell P, Liss P (2005). Pathophysiology of contrast medium induced nephropathy. Kidney Int.

[35] Pierson-Marchandise M, Gras V, Moragny J, Micallef J, Gaboriau L, Picard S (2017). The drugs that mostly frequently induce acute kidney injury: as case—noncase study of a pharmacovigilance database. Br J Clin Pharmacol.

[36] Luther MK, Timbrook TT, Caffrey AR, Dosa D, Lodise TP, LaPlante KL (2018). Vancomycin plus piperacillin-tazobactam and acute kidney injury in adults: a systematic review and meta-analysis. Crit Care Med.

[37] Sjövall J, Westerlund D, Alván G (1985). Renal excretion of intravenously infused amoxycillin and ampicillin. Br J Clin Pharmcol.

[38] Fogazzi GB, Cantù M, Saglimbeni L, Daudon M (2003). Amoxycillin, a rare but possible cause of crystalluria. Nephrol Dial Transplant.

[39] Zeller V, Puyraimond-Zemmour D, Sené T, Lidove O, Meyssonnier V, Ziza JM (2016). Amixicillin crustalluria, an emerging complication with an old and well-known antibiotic. Antimicrob Agents Chemother.

[40] Chien CC, Wang HY, Wang JJ, Kan WC, Chien TW, Lin CY (2011). Risk of acute kidney injury after exposure to gadolinium-based contrast in patients with renal impairment. Ren Fail.

