# Supplementary material for: Nephrotoxic drug burden among 1001 critically ill patients: impact on acute kidney injury
Source: Ann Intensive Care. 2019 Sep 23;9:106. doi: 10.1186/s13613-019-0580-1 (PMC6757082; doi:10.1186/s13613-019-0580-1)
Supplement: Supplementary file 1 — Additional file 1: Figure S1. Acute kidney injury (AKI) worsening cases and control matching. Table S1. Main characteristics of matched and unmatched patients. Table S2. Relative contribution individual drugs to the difference in nephrotoxic burden experienced by cases and controls. [file 13613_2019_580_MOESM1_ESM.docx]

**Nephrotoxic prescription burden among 1,001 critically ill patients: impact on acute kidney injury**

Stephan EHRMANN, Aurélie JORET, Julie HELMS, Laurent MARTIN-LEFEVRE, Nicolas MENUNIER-BEILLARD, Jean-Etienne HERBRECHT, Dalila BENZEKRI-LEFEVRE, René ROBERT, Arnaud DESACHY, Fréderic BELLEC, Gaëtan PLANTEFEVE, Jean-Pierre QUENOT, Jean-Claude LACHERADE, Ferhat MEZIANI, Bruno GIRAUDEAU, Elsa TAVERNIER, Pierre-François DEQUIN, Clinical research in intensive care and sepsis – Trial group for global evaluation and research in sepsis (CRICS-TRIGGERSEP network)

**Additional file 1**

KDIGO classification: the creatinine criterion of the classification was evaluated based on daily serum creatinine concentrations compared to the patient baseline serum creatinine concentration. Baseline serum creatinine was defined as a serum creatinine concentration measurement less than 1 year before inclusion outside of any AKI episode. This information was available for 568 patients (57%). In case of unavailable data, for patients without chronic kidney disease, baseline serum creatinine concentration was estimated using the Modification of Diet in Renal Disease (MDRD) equation [E1], assuming a glomerular filtration rate of 75 mL/min/1.73m^2^ this calculation could be performed for 420 patients (42%) For patients with chronic kidney disease, baseline serum creatinine concentration, if not available in the patient’s chart, was assumed to be the lowest value observed during the hospital stay (this baseline value was used for only 13 patients (1%). The diuresis criteria of the KDIGO classification was evaluated based on oliguria quantified in mL/kg, only in patients equipped with a urinary catheter, based on baseline patients’ weight or, if unavailable, the first weight measured in the intensive care unit (ICU).

Figure S1: Acute kidney injury (AKI) worsening cases and control matching


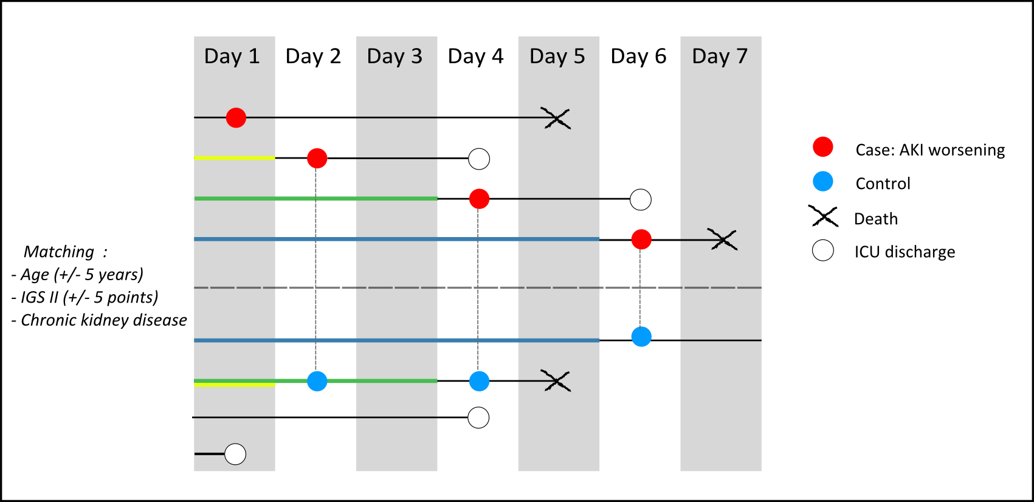


Cases were patients admitted to the ICU without AKI (KDIGO stage 0), who subsequently developed KDIGO stage 1, 2 or 3 AKI and patients admitted with already ongoing AKI (KDIGO stage 1 or 2), who subsequently developed KDIGO stage 2 and/or 3 AKI. Controls were patients without AKI worsening a given day during the first seven days of the ICU stay (i.e. no AKI or same KDIGO stage as at admission). Each individual patient could only serve either as a case or as a control but never both. Cases were matched 1:1, on age (5 years margin), presence of chronic kidney disease and admission simplified acute physiology score II (SAPS2) (5 points margin), with control patients alive the index day the case patient developed AKI worsening.

| Table S1: Main characteristics of matched and unmatched patients | Cases (n=327) | Controls (n=327) | Unmatched cases (n=24) |
| --- | --- | --- | --- |
| Female gender | 108 (33%) | 133 (41%) | 11 (46%) |
| Age | 67±14 | 67±13 | 73±12 |
| Main admission diagnosis |  |  |  |
| De novo acute respiratory failure  Coma, seizure  Sepsis and septic shock  Chronic respiratory failure exacerbation  Cardiac arrest  Hemorrhagic and hypovolemic shock  Cardiogenic shock  Post-operative monitoring  Acute renal failure  Other | 56 (17%)  50 (15%)  50 (15%)  49 (15%)  34 (10%)  9 (3%)  10 (3%)  14 (4%)  2 (1%)  53 (16%) | 74 (23%)  50 (15%)  45 (14%)  53 (16%)  27 (8%)  11 (3%)  8 (2%)  1 (0%)  3 (1%)  55 (17%) | 5 (21%)  3 (13%)  3 (13%)  2 (8%)  7 (29%)  0  0  0  0  4 (17%) |
| Simplified acute physiology score II | 50±17 | 50±16 | 71±20 |
| Co-morbidities |  |  |  |
| Arterial hypertension  Ischemic heart disease  Chronic heart failure  Peripheral artery disease  Chronic respiratory failure  Cirrhosis  Chronic kidney disease  Diabetes mellitus | 196 (60%)  61 (19%)  50 (15%)  43 (13%)  74 (23%)  20 (6%)  26 (8%)  88 (30%) | 157 (48%)  54 (17%)  45 (14%)  77 (7%)  71 (22%)  18 (6%)  26 (8%)  72 (22%) | 17 (71%)  7 (29%)  3 (13%)  6 (25%)  3 (13%)  4 (17%)  17 (71%)  8 (33%) |
| At least one nephrotoxic drug during the first 7 days in the ICU | 237 (72.5%) | 192 (58.7%) | 16 (66.7%) |
| At least one nephrotoxic drug within 48 hours before ICU admission | 230 (70.3%) | 210 (64.2%) | 19 (79.2%) |
| Catecholamine infusion | 189 (75.3%) | 140 (78.7%) | 17 (81.0%) |
| Invasive mechanical ventilation | 253 (77.4%) | 182 (55.7%) | 22 (91.7%) |
| Qualitative variables are presented as count (percentage) and quantitative variables as mean±standard deviation | | | |

Patients serving as cases and controls had similar characteristics except a high proportion of female in the control population, more de novo acute respiratory failure and less cardiac arrest. 24 cases (7% of cases) could not be adequately matched with controls. Those patients exhibited a more advanced age, more frequent admission for cardiac arrest, a high incidence of comorbidities and a high SAPSII, all pointing towards a very specific population with very poor outcome.

| Table S2: Relative contribution individual drugs to the difference in nephrotoxic burden experienced by cases and controls | | | |
| --- | --- | --- | --- |
|  | Control | Cases | Difference |
| Iodinated contrast media | 0.09±0.3 | 0.2±0.4 | 0.11 |
| Diuretics | 0.2±0.5 | 0.4±0.9 | 0.2 |
| Loop diuretics | 0.2±0.5 | 0.4±0.9 | 0.2 |
| Thiazide diuretics | 0.01±0.2 | 0.02±0.2 | 0.01 |
| Potassium sparing diuretics | 0.01±0.1 | 0.009±0.2 | -0.001 |
| Antibiotics | 0.3±0.7 | 0.3±0.7 | 0 |
| Vancomycin | 0.1±0.4 | 0.1±0.6 | 0 |
| Gentamycin | 0.05±0.3 | 0.03±0.2 | -0.02 |
| Amikacin | 0.06±0.3 | 0.10±0.4 | 0.04 |
| High dose betalactam | 0.04±0.3 | 0.06±0.3 | 0.02 |
| Sulfamethoxazole trimethoprim | 0.02±0.2 | 0.02±0.2 | 0 |
| Rifampicin | 0.02±0.2 | 0.03±0.3 | 0.01 |
| Total nephrotoxic burden | 0.86±1.3 | 1.20±1.76 | 0.34 |
| Mean±SD nephrotoxic burden among the 327 matched cases and control patients is indicated in drug.days (see text). Difference denotes the difference between mean burden among cases and controls. Iodinated contrast media, loop diuretics and amikacin account for most of the difference in nephrotoxic burden observed between cases and controls. | | | |

References:

S1. Levey AS, Bosch JP, Lewis JB, Greene T, Rogers N, Roth D (1999) A more accuratemethod to estimated glomerular filtration rate from serum creatinine: a new prediction equation. Modification of diet in renal disease study group. Ann Intern Med 130:461-470
